# Predicting the drift of small cetaceans stranded along the Atlantic coast of the Iberian Peninsula: Parametrization of the MOTHY drift model

**DOI:** 10.1371/journal.pone.0315593

**Published:** 2024-12-17

**Authors:** Camille Deslias, Pierre Daniel, Alfredo López, José Martínez-Cedeira, Vincent Ridoux, Hélène Peltier

**Affiliations:** 1 Centre d’Études Biologiques de Chizé-La Rochelle, UMR 7372 CNRS, La Rochelle Université, Chizé, France; 2 Observatoire Pelagis, UAR 3462 CNRS, La Rochelle Université, La Rochelle, France; 3 Météo-France, Direction des Opérations pour la Prévision, Département Prévision Marine et Océanographique, Toulouse, France; 4 CEMMA Coordinadora para o Estudo dos Mamíferos Mariños, Nigrán, Spain; 5 Departamento de Biología & CESAM, Universidade de Aveiro, Aveiro, Portugal; Central Marine Fisheries Research Institute, INDIA

## Abstract

Marine mammal populations, particularly the common dolphin *Delphinus delphis* in the North-East Atlantic, play an essential role as indicators of ecosystem health. Effective monitoring of these populations is essential for assessing anthropogenic impacts, especially in the context of current threats such as fisheries bycatch. The MOTHY drift model, initially designed for oil spills and then adapted to carcass drift, is being used in part of the North East Atlantic (Bay of Biscay, English Chanel, and North Sea) to estimate the bycatch mortality of common dolphins. This study presents the parametrization of the drift model to estimate the bycatch mortality of common dolphins in the Iberian Peninsula waters. By comparing the actual stranding location of tagged dolphin carcasses off the Galician coast with their stranding location predicted by the drift model, we determined the best setting for the environmental input parameters. The results reveal that a 4 arc-minutes bathymetry resolution, coupled with consideration for currents, optimally predicts stranding locations in the Iberian Peninsula coast. The model’s accuracy in predicting stranding locations is 18.25 ± 14.77 km. This adaptation not only contributes to the ongoing assessment of the impacts of bycatch on common dolphin populations in the Iberian Peninsula, but also provides a standardized methodology for estimating bycatch mortality at the population level. This work can also be used as a basis for further applications for other small cetacean species in wider distribution areas, supporting comprehensive population-level assessments and management strategies.

## Introduction

Continuous monitoring of marine ecosystems is essential to the proper assessment of the impact of human activities at sea. In this context, marine mammals are valuable indicators of ecosystem changes [1] both from the top-down, through the signals present in their tissues, and from the bottom-up, through their distribution patterns and movements at large scale [2]. To monitor marine mammal populations, it is necessary to obtain demographic parameters that describe the population, including population size and mortality [3, 4]. To measure these parameters effectively we need to determine the most relevant monitoring area for the population, variously named assessment, conservation or management units. Marine mammals are typically highly mobile species living over large areas at fairly low densities, making it difficult to define population entities and collect the data on structure and abundance expected at the right geographical scale [5].

Anthropogenic removal from a population with a slow life history, such as small cetaceans, can have a major impact on its dynamics. With high investment in growth and high costs of offspring rearing [6], small cetaceans have low maximum population growth rates, resulting in a poor demographic resilience. Therefore, they are particularly vulnerable to population decline when human activities result in additional mortality [7]. Fisheries bycatch is known to be the first source of anthropogenic mortality for small cetaceans worldwide [8] and plays a significant role in the decline of many populations, such as the harbor porpoise *Phocoena phocoena* in the Baltic Sea, the Commerson’s dolphin *Cephalorhynchus commersonii* in Argentinian waters, the Burmeister’s porpoise *Phocoena spinipinnis* in Peru and the vaquita *Phocoena sinus* in the Gulf of Mexico [9–12].

The common dolphin *Delphinus delphis* is an abundant small cetacean found in all oceans, ranging from tropical to temperate waters. In the North-East Atlantic, the species is distributed both in oceanic waters and on the continental shelf up to about 60°N [13]. It is commonly agreed that all common dolphins inhabiting the North-east Atlantic area belong to the same sub-population, even though uncertainty remains as to possible connections with common dolphins living further west or south, and possible substructure supported by ecological tracers [14–17]. An assessment unit has been established to implement consistent indicators and methodologies to monitor the North East Atlantic population [14]. The area covers OSPAR Regions II (Greater North Sea), III (Celtic Sea), and IV (Bay of Biscay and Iberian coast) [18]. In this assessment unit, the abundance of common dolphins was estimated at 634,286 individuals (CV = 0.307; 95% CI: 352,227–1,142,213) in the summer of 2016 from the Observe program and SCANS-III aerial and shipboard surveys [17]. This number includes both common dolphins and unidentified dolphins (probably common dolphins or striped dolphins (*Stenella coeruleoalba*) [17]. Another estimate for the same year, focusing solely on common dolphins and based on campaign data (excluding observational programs), indicated a population of 467,673 individuals (95% CI: 281,100–778,000) [19]. More recently, following the same methodology, a total of 439,212 common dolphins (95% CI: 309,153–623,987) was estimated during the summer of 2022 from SCANS-IV aerial and shipboard surveys [20].

With the aim of assessing the conservation status for this sub-population, it is essential to estimate anthropogenic mortality levels at the population scale, especially mortality by bycatch. Two methods are used. The first method consists in estimating bycatch mortality by implementing on-board observer programs or remote digital monitoring systems [21]. Remote digital monitoring, like on-board cameras, is considered as the least biased monitoring system, followed by dedicated observer programs, while multitasked fishery observer programs are considered the most biased [22]. On-board cameras offer potential solution but faces many challenges, primarily due to a large majority of fishermen being reluctant to equip their boats. This reluctance stems from a strong perception of intrusion into their private lives and a deep mistrust regarding how the data will be used [21, 23].

The second approach is to use stranding data series to estimate total bycatch mortality by using a drift model [24]. The drift model MOTHY (*Modèle Océanique de Transport d’HYdrocarbures*), initially designed for modelling the drift of oil slicks and floating objects of interest to maritime safety operators [25, 26], has been adapted to predict the drift of small cetaceans [27]. Based on stranded animals with bycatch marks and environmental drivers, the model can estimate the areas where they most likely died and the total number of animals that died at sea taking carcass buoyancy and stranding probability into account. Since 2019, this method has been used by the Working Group on Marine Mammal Ecology (WGMME) and the Working Group on Bycatch Protected Species (WGBYC), which meet every year under the International Council for the Exploration of the Sea (ICES), as a complement to on-board observations [28]. These estimates were also incorporated into the work of the ICES working group aimed at evaluating the emergency measures requested by Non-Governmental Organizations (NGO) to the European Commission. This work provided estimates of common dolphin bycatch in the Bay of Biscay, in order to evaluate the relevance of the measures requested by NGOs, and to propose scenarios for fishery closure to meet different conservation objectives [29, 30]. Additionally, this method offers spatial and temporal representations of bycatch events, identifying probable mortality locations. When combined with other data sources, this tool provides valuable insights for decision-makers and stakeholders [31]. This second method of bycatch mortality estimation is used in the Bay of Biscay, as well as the English Channel and the North Sea, three subregions of the assessment unit. However, the mortality of common dolphins in the entire Iberian Peninsula is less well known. Incidental catches in the area are known from strandings and interviews with fishermen in Galicia and Portugal [8, 32–35]. Some estimates have focused on specific areas and fisheries, such as pairtrawler in northern Spain [36], or based on data from on-board observer programs [30]. To date, the bycatch estimates of the fishing industry covering the entire Atlantic region west of the Iberian Peninsula are lacking.

To estimate mortality based on stranding data in the Iberian Peninsula and, hence have a common approach for estimating and assessing bycatch across most of the OSPAR Region, it is proposed to adapt the method used in the Bay of Biscay, the English Channel, and the North Sea to the Iberian Peninsula. This would be an important step toward the assessment of bycatch mortality over the whole common dolphin assessment unit with a standardized methodology. A common indicator would provide a more comprehensive and consistent understanding of bycatch mortalities and their impact on the North-East Atlantic subpopulation of common dolphins, to implement effective management and conservation measures. Therefore, the main objective of the present work is to adapt the drift model to a new area by selecting the most appropriate setting for calculation methods and environmental parameters following the principles initially developed in the Bay of Biscay, the English Channel and the North Sea [27].

## Materials and methods

### Study area

**Fig 1:**
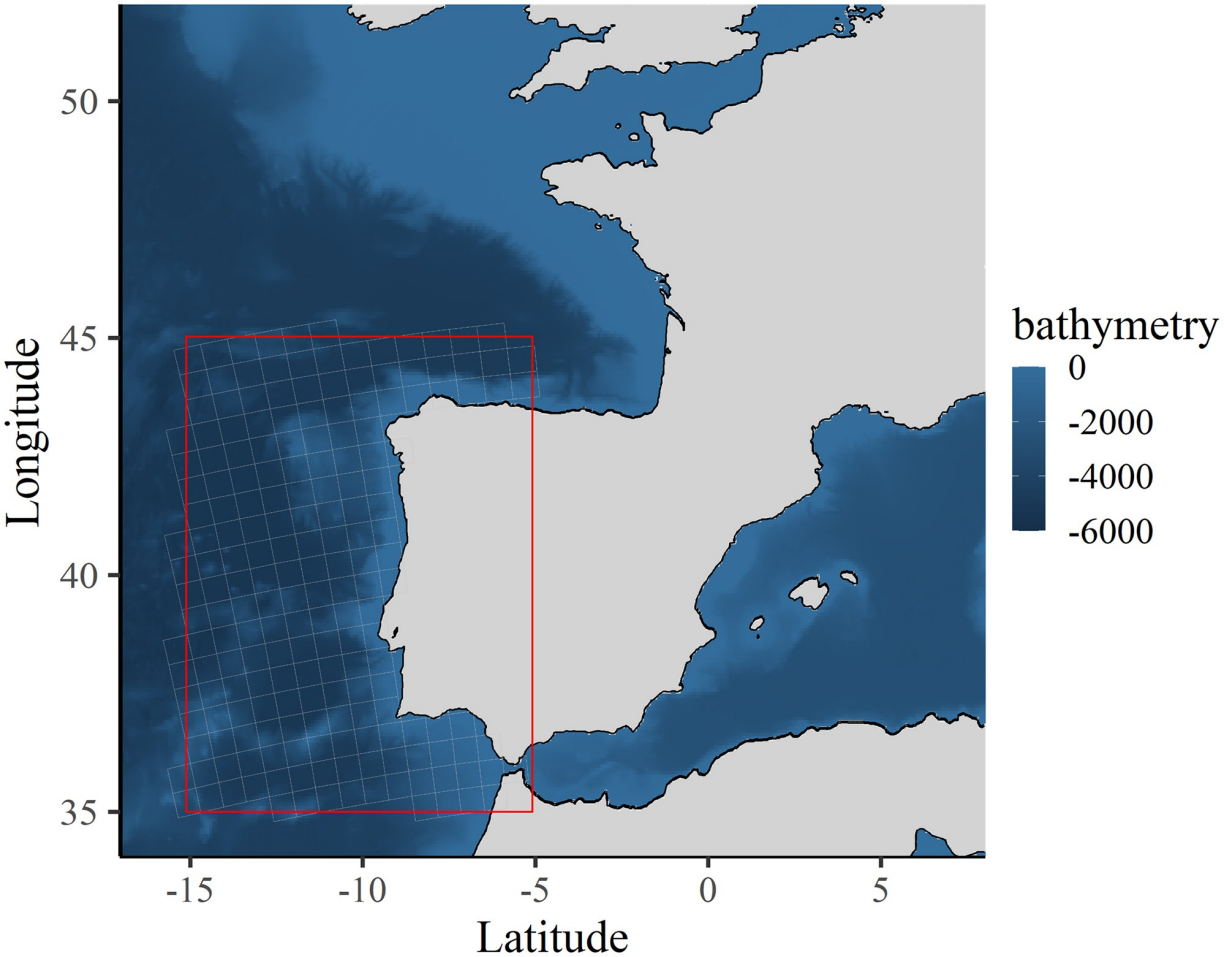
Boundary of the study area (in red) covering the coasts of the western Iberian Peninsula.

The study area covers the Atlantic waters of the Iberian Peninsula along the Portuguese mainland coast and the north-western Spanish coast. It includes the coasts of Algarve, Alentejo, Center and North of mainland Portugal, and Galicia in Spain. The continental shelf is relatively narrow, 40 to 50 km wide off Portugal and 30 km wide further north [37]. Overall, the coastline is dominated by rocky coasts, interrupted by extensive sandy beaches in northern Portugal and in the eastern part of Algarve. In northwest Spain, the Galician coast features a variety of landscapes, including sandy beaches, rocky platforms, and cliffs [38]. The study area, which extends from 35°N to 45°N and from 15°W to 5°W (Fig 1), is divided into 195 statistical squares of equal size (60 km x 60 km). All maps were generated using the ggplot2 package (version 3.5.1), the sf package (version 1.0.14), and the marmap package (version 1.0.10) to extract bathymetric data from the NOAA database, in R (version 4.2.1; R Core Team, 2022).

### General experimental design

**Fig 2:**
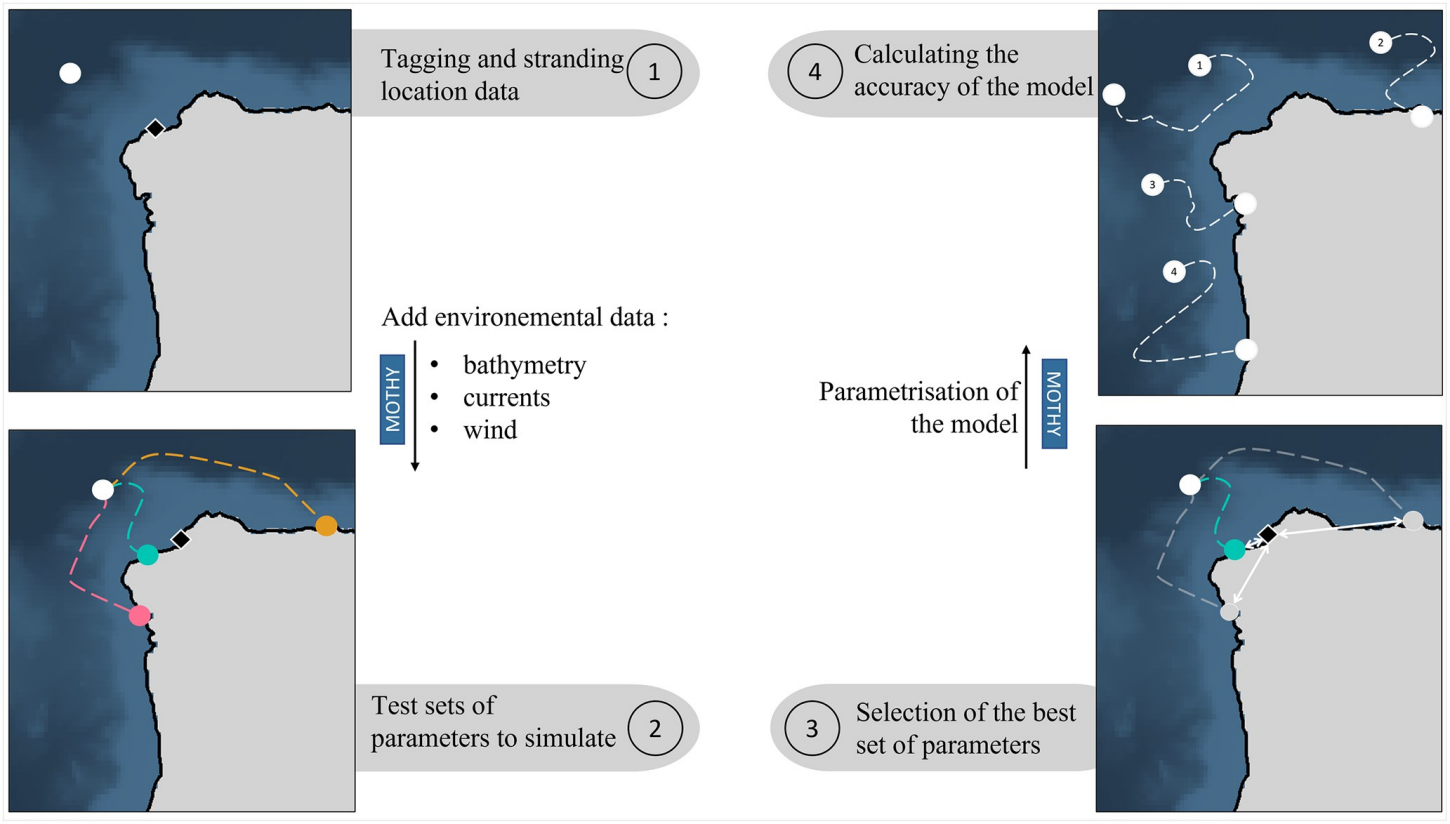
Schematic representation of the experimental strategy. The ‘MOTHY’ box indicates where the drift model was used in the process (between steps (1) and (2), and between steps (3) and (4)). The white dots represent the tagging location, and the black diamond represents the actual stranding location. The dashed lines represent the trajectory of simulated drifts, (2) with each color representing a set of parameters. (3) Points and dashed lines in grey represent the trajectoires simulated with unselected parameter sets. The distance between the predicted and the actual stranding locations is indicated by white diamonds. (4) Numbers correspond to tagged carcasses with the drift trajectory simulated by MOTHY paramatrised.

Common dolphin bycaught, tagged dead by fishermen, released at sea, and recovered stranded were used in order to validate the parameters that are the best suited to predict dolphin carcass drift and stranding off the Atlantic coast of the Iberian Peninsula. The method is divided into four stages (Fig 2) following the strategy implemented earlier in the Bay of Biscay [24]. The first stage involves collecting data from tagged dolphin carcasses, including the date, time and location of the tagging and the location of the stranding of the tagging carcass (1). The second stage involves simulating the drift trajectories of tagged and stranded dolphins carcasses by testing various environmental parameters (different bathymetry accuracy, presence or absence of low-frequency currents) and computation option (computational time) (2). The set of parameters that best simulates the trajectory closest to reality for the tagged carcasses are then selected (3). Finally, the trajectories of all the tagged carcasses are simulated to determine the accuracy of the model in predicting the stranding location of a carcass (4).

### Tagging and stranding location data

**Fig 3:**
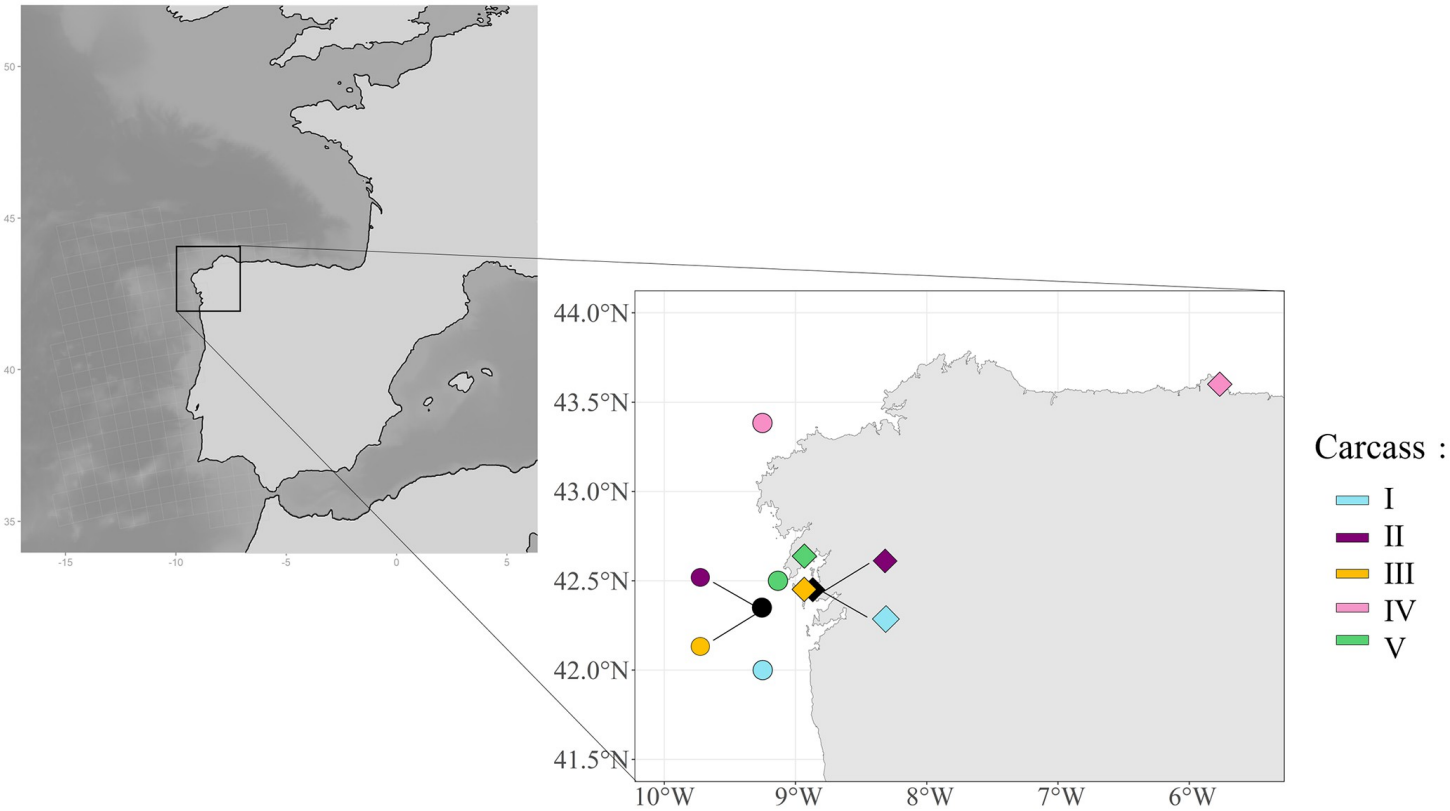
Tagging and stranding locations of the five bycaught dolphins in 2009 and 2010 in Galicia (Spain). Release locations are represented by circles and stranding locations are represented by diamonds. Color codes represent specific individuals. The black symbols represent the location of two overlapping individuals.

Off the coast of Galicia, Coordinadora para o Estudo dos Mamiferos MArinos (CEMMA), in collaboration with fishermen, tagged bycaught cetaceans between 2007 and 2012. Bycaught cetaceans, primarily common dolphins, were recovered dead on board and tagged with plastic rings bearing a unique identifier. For each individual, the species, size, date of capture, and type of fishing gear used were recorded. The cetaceans were then released at sea with their GPS location noted at the time of release. Bycatches were observed in three types of fishing gears: pair trawls, gillnets, and trammel nets. Of the 24 dolphins tagged, only five individuals were found stranded on the coast in 2009 and 2010, which were used for analysis (Fig 3).

### Presentation of the drift prediction model

The drift prediction model MOTHY was developed by MétéoFrance, the French national meteorological agency. MOTHY is an integrated system that includes hydrodynamic coastal ocean modelling and real time atmospheric forcing from a global or limited area model. The aim was initially to predict the trajectory of oil slicks or floating containers to inform maritime safety operator [25]. This model can predict the drift of floating objects considering different forcing parameters such as tides, winds and currents. MOTHY integrates high-frequency components of currents driven by wind and tide with low-frequency currents associated with large-scale ocean circulation. The high-frequency component is modeled through an integrated hydrodynamic model connected to an advanced Ekman-type scheme. The low frequency component is sourced from operational oceanography system, using currents obtained at a fixed depth representative of the mean depth of the Ekman layer, with a technique similar to that described in [39]. The model was adapted to small cetaceans in the Bay of Biscay, the English Channel, and the North Sea [24] by adjusting the buoyancy rate and the wind resistance of the carcass. Winds were provided by the European Centre for Medium-Range Weather Forecasts via MétéoFrance, at 6-hourly resolution. The ocean currents data used in this study have a daily temporal resolution and a spatial resolution of 0.083°. These data are extracted from the **GLOBAL_MULTIYEAR_PHY_001_030** product of the Mercator Ocean model, available on the Copernicus Marine Environment Monitoring Service website. This product is derived from the Operational Mercator global ocean analysis and forecast system at a 1/12 degree resolution, validated by observations. It provides long-term reanalysis from January 1, 1993, to May 28, 2024, making it particularly suitable for climate studies. Bathymetry data for the Iberian coast is provided by the General Bathymetric Chart of the Ocean at resolutions ranging from 1 arc-minute to 5 arc-minutes.

Two simulation directions are available. The direct drift consists of having an initial location at sea on day named *D*_0_, and an endpoint on day named *D*_1_ with *D*_1_ = *D*_0_ + *number of drift days*. This direction can be used to determine where a dolphin bycaught at sea should strand. The reverse drift consists in starting from a stranding point on land on day *D*_1_ and obtaining the initial location on day *D*_0_ with *D*_0_ = *D*_1_ − *number of drift days*. The aim is to find the origin of stranded animals. Two methods of calculation are available: either a deterministic method or a probabilistic method. The deterministic approach predicts one drift at a time, so only one trajectory is predicted for each cetacean. The probabilistic approach predicts 8671 drifts for each cetacean with a combination of different buoyancy rates, floating behavior and surface wind.

In this study we use the direct drift direction with the deterministic approach.

### Parameters selection

For each dolphin, the carcass drift was simulated, using direct drift, with the initial point of the drift was the release location at sea. Five bathymetry data spatial resolutions were tested, ranging from the finest to the most degraded resolution (from 1 to 5 arc-minutes). The resolution of the bathymetry influences the drifts made in shallow waters (< 100m) by taking better account of the details that influence currents. The inclusion of low-frequency currents in drift modeling was also evaluated (simplified by the term “currents” for the rest of the analysis). Wind and surface currents are systematically taken into account in the simulations.

From those, the set of parameters that predict the most realistic location of the stranding was selected based on two criteria. The first criterion was the computational time, which can be a limit when large numbers of drifts are calculated. The second criterion was the distance between the real and the predicted stranding location for each individual. Based on the fact that the work is carried out on a regional scale, it is assumed that when the distance is less than 10 km, the pair of parameters can be selected as having an accurate prediction. In the event that the distances exceed 10 km for all combinations, the combinations associated with the three shortest distances are selected. If the drift simulation did not result in stranding on shore, then the distance between the predicted stranding and the real stranding could not be calculated and was indicated as N/A. A Kruskal-Wallis test was conducted to evaluate the differences in measured distances among the parameter groups identified as the most accurate. This choice is justified by the small sample size, which necessitates the use of a non-parametric test to ensure reliable results.

### Drift simulations of all tagged dolphin carcasses

The direct drift of the 24 tagged carcasses was predicted over 25 days using the best set of parameters. Simulations are based on the assumption that all dolphins would float and that if they reach the coast they would be reported to the local authorities. This analysis focuses on two different aspects: firstly, the number of dolphins predicted to be stranded that are indeed found ashore; secondly, the total number of dolphins predicted to be stranded, whether or not they are eventually found. In other words, we examine both the accuracy of the model’s predictions by comparing observed results with positive predictions, and the overall coverage of the model by considering all predicted cases, whether confirmed by observation or not.

### Ethics statement

This work reports new results that have not been previously reported and will not be reported elsewhere. This work has been carried out in accordance with the European regulation on the use of stranded dead cetaceans for scientific and conservation purposes. The authors have therefore followed the general guidelines for the ethical use of animals in research, the legal requirements in Europe. No live animals were used in this study, only dead cetaceans bycatch and/or found stranded along Galician coast in Spain. No samples were used in this study. The administrative authorisation for the handling of specimens stranded or rescued off the coast of Galicia, the collection and preservation of their biological samples and the performance of necropsies for scientific and conservation purposes was granted by the Spanish Ministry of Ecological Transition and Demographic Challenge.

## Results

### Parameters selection

The first criterion, the computational time, strongly depends on the accuracy of the bathymetry. The use of bathymetry with a resolution of 1 arc-minute leads to trajectory calculation times ranging from 4h45 to 7h25 (Table 1). These calculation times seem too high for a large-scale application of this model (i.e. simulation of several hundred trajectories). It will therefore not be considered as a potential parameter of the model. Using a resolution of 2 arc-minutes, the calculation time is between 25 minutes to 46 minutes. Bathymetric resolutions 3, 4 and 5 arc-minutes have calculation times shorter than 10 minutes. The shortest computational time, between 1 minute and 1 minute 30, corresponds to the least precise bathymetry (5 arc-minutes).

**Table 1:** Simulated trajectory characteristics of five common dolphin carcasses (I, II, III, IV, V) tagged in 2009 and 2010.

2009					
Bathymetry resolution	1 arc-minute	2 arc-minutes	3 arc-minutes	4 arc-minutes	5 arc-minutes
with currents					
Computational time	7:25:00	0:40:00	0:08:00	0:03:30	00:01:30
Distance real/ predicted stranding	I. 20 km II. 9 km III. 10 km	I. 25 km II. 9 km III. 10 km	I. 17 km II. 20 km III. 1 km	I. 23 km II. 5 km III. 8 km	I. 16 km II. 5 km III. 8 km
without currents					
Computational time	7:00:00	0:46:00	0:06:30	0:03:00	00:01:30
Distance real/ predicted stranding	I. 18 km II. 10 km III. 11 km	I. 18 km II. 10 km III. 11 km	I. 19 km II. 17 km III. 19 km	I. 23 km II. 6 km III. 1 km	I. 18 km II. 11 km III. 11 km
2010					
with currents					
Computational time	5:10:00	0:25:00	0:04:45	0:02:00	00:01:00
Distance real/ predicted stranding	IV. N/A V. N/A	IV. N/A V. N/A	IV. N/A V. 27 km	IV. N/A V. 37 km	IV. N/A V. N/A
without currents					
Computational time	04:45:00	0:25:00	0:05:00	0:02:00	00:01:00
Distance real/ predicted stranding	IV. N/A V. 25 km	IV. N/A V. 25 km	IV. N/A V. 99 km	IV. N/A V. 37 km	IV. N/A V. 181 km

**Fig 4:**
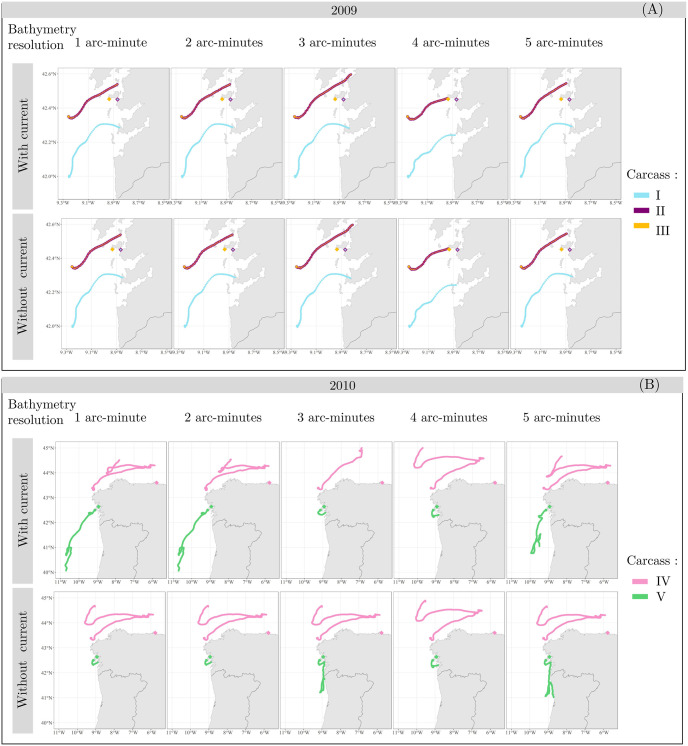
Map of the simulated drift trajectory of five common dolphins tagged in 2009 (A) and 2010 (B). Different bathymetry resolutions and the presence or absence of currents are tested in the model parameterization. The black diamond represents the location where the carcass was marked. The actual stranding site is represented by a coloured diamond shape.

The computational time line corresponds to the time taken to calculate all strandings. The distance between actual and predicted stranding is given in kilometers for each carcass. Simulated trajectories of the carcasses are shown in Fig 4.

The second selection criterion was the distance between the real and the predicted stranding location for each individual. The wind and surface currents is always considered in the model by default. As mentionned above, the inclusion or not of the currents refers to low frequency currents. The trajectories of carcass IV are excluded from the selection of parameters because the drift simulation never led to a stranding (Table 1, Fig 4B).

In the case of carcass I, the three shortest distances between the real and predicted stranding are 16 km, 17 km and 18 km. Consequently, the combinations selected are the bathymetric resolution of 2 arc-minutes without the addition of currents, the bathymetric resolution of 3 arc-minutes without currents and the bathymetric resolution of 5 arc-minutes with and without the addition of currents. The trajectory of carcass II is more accurately predicted with a 4 arc-minutes resolution, both with and without currents, and with 5 arc-minutes resolution with currents (Table 1, Fig 4A). The trajectory of carcass III is more accurately predicted with a 3 arc-minutes resolution, considering the addition of currents, and with a 4 arc-minutes resolution without the addition of currents. Predictions using 4 and 5 arc-minute resolution bathymetry and the addition of currents also remain satisfactory in terms of accuracy for the trajectory of carcass III (Table 1, Fig 4A). In the case of carcass V, the three shortest distances between the real and predicted stranding are 25 km, 27 km and 37 km. Consequently, the combinations selected are the 2 arc-minutes resolution without currents, a 3 arc-minutes resolution with currents, and a 4 arc-minutes resolution both with and without currents (Table 1, Fig 4B).

When comparing the results for each carcass, several sets of parameters demonstrated consistency across three trajectories (Table 2). The parameter pairs with a bathymetric resolution of 5 arc-minutes and currents were selected for carcasses I, II, and III; however, they failed to simulate the stranding of carcass V. As a result, this set of parameters is not considered a valid option. Conversely, the sets of parameters with a bathymetric resolution of 4 arc-minutes, both with and without currents, showed consistency for the trajectories corresponding to carcasses II, III, and V. Additionally, the set of parameters with a 3 arc-minute resolution and currents is retained for carcasses I, III, and V (Table 2). The Kruskal-Wallis test revealed that there was no significant difference in the distances between the sets of parameter (Kruskal-Wallis chi-squared = 0.126, df = 2, p = 0.939). These results suggest that the use of these sets of parameter does not significantly influence the distances between simulated and actual stranding locations. The bathymetric resolution of 4 arc-minutes is favored over 3 arc-minutes due to its faster computational time (Table 1). Considering that the use of currents did not significantly affect accuracy or calculation time, and given the small sample size, the decision has been made to use 4 arc-minutes bathymetry resolution data together with currents as the input parameters for MOTHY in the Iberian region. The inclusion of currents is motivated by their potential impact on other trajectories in the study area, beyond the sampling region considered here.

**Table 2:** Summary of the results for each carcass drift (I, II, III, and V) to select the best parameters to fit the model.

Bathymetry resolution		2 arc-minutes		3 arc-minutes		4 arc-minutes		5 arc-minutes	
Currents		With	Without	With	Without	With	Without	With	Without
Carcass	I	✕	✓	✓	✕	✕	✕	✓	✓
	II	✓	✕	✕	✕	✓	✓	✓	✕
	III	✕	✕	✓	✕	✓	✓	✓	✕
	V	N/A	✓	✓	✕	✓	✓	N/A	✕

The check mark means that the parameters were selected as more accurate. N/A means that the model with its parameters failed to predict the stranding of the carcass.

### Drift simulations of tagged dolphin carcass

**Fig 5:**
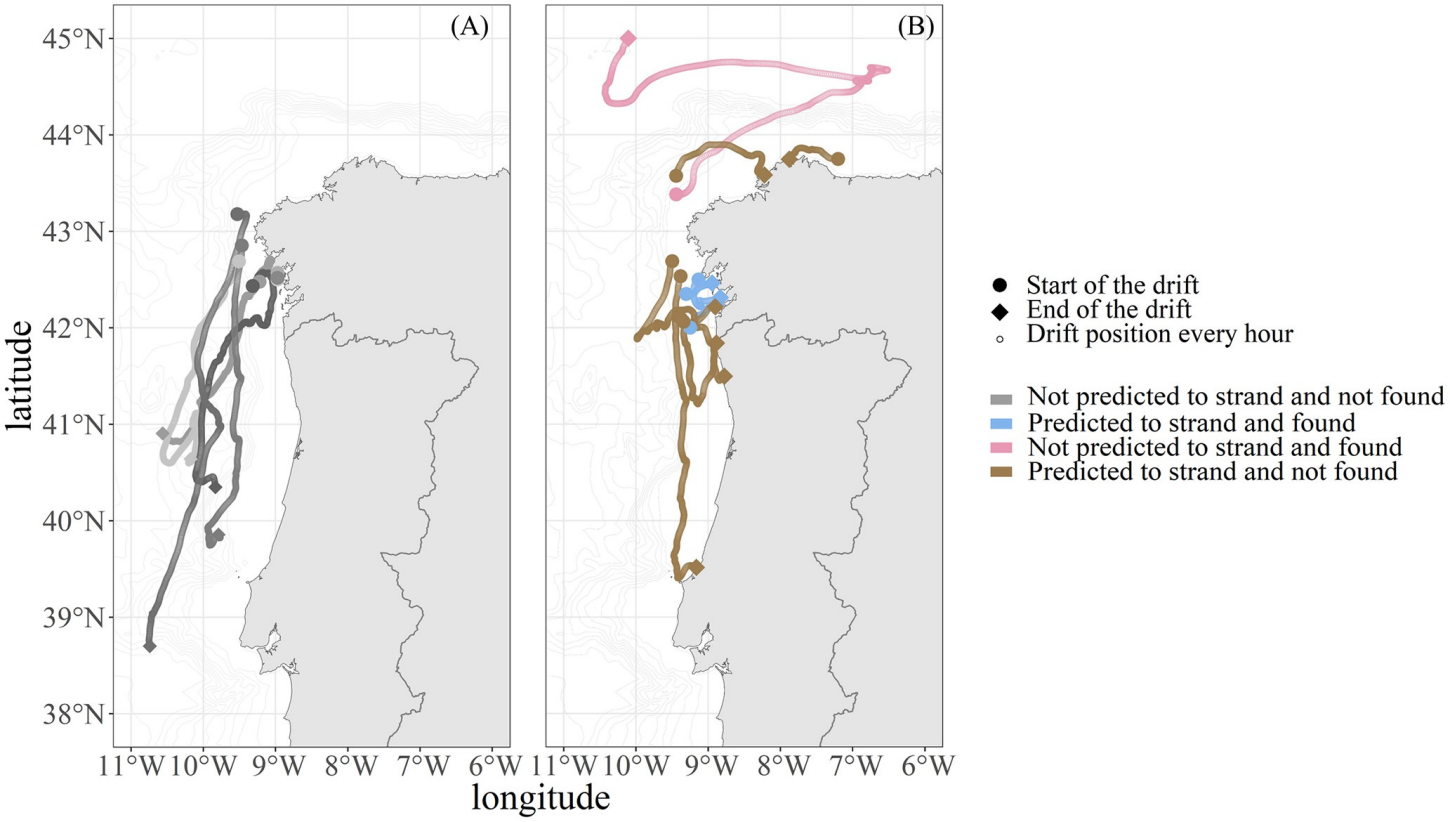
Trajectory of the drift simulation of 24 tagged dolphin carcasses. The start of the trajectory is represented by a circle. The end of the trajectory is represented by a diamond. (A) The trajectories in grey were not predicted to become stranded and were not found on shore. (B) The trajectory of carcasses predicted to become stranded and found on the coast (in blue), not predicted to become stranded and found stranded (in pink) and predicted to become stranded and not found (in brown). The position of the stranding found of the pink trajectory is available in Figs 3 and 4 (carcass IV).

Of the 24 tagged individuals, stranding was predicted for 10 carcasses after 25 days of drift (Fig 5). Of the five individuals found on-shore, four carcasses were predicted by the model to be stranded (in blue). A total of six carcasses were predicted to be stranded but were not detected by the stranding networks (in brown). Finally, only one carcass predicted not to strand was found stranded (in pink). The average time between the location of release at sea and the stranding location was 10.43±10.45 days. The distance between the real stranding location and the predicted stranding location was 18.25±14.77 km (Table 1).

## Discussion

The tests carried out on the drifts of the five tagged carcasses of common dolphins allowed us to determine that the 4 arc-minutes resolution bathymetry and the use of currents are the best combination among those tested in the Iberian Peninsula. Although the use of currents did not have a significant effect on the trajectories of these five individuals, we believe that in some cases the currents could influence the drift of a floating carcass in the whole area. Around Spain and Portugal, the currents derive from the circulation of the North Atlantic gyre, with a current that can carry the carcasses southwards along Galicia and Portugal, and a current that carries them eastwards along the Spanish coast of the Bay of Biscay. By choosing to keep the currents in the model parameters, we expect to be closer to reality in most cases.

A higher number of tagged dolphins could highlight other combinations of input parameters. Given the accessibility of the data, a long-term effort is required to finalize the model. For instance, the model in the Bay of Biscay was first fitting from about 100 dolphins tagged on fishing boats, and is updated every year over 10 years [24]. In 2010, the trajectory of the carcass IV can not be used because the predicted trajectory was too far from the actual stranding location. This is the most northerly released individual in Galicia and no other dolphins were tagged in this area to compare whether this was due to specific environmental conditions in this area, or other factors. At this stage, we consider the carcass IV as a false negative, indicating that the model can be further improved. A larger number of tags will also allow us to measure the sensitivity and accuracy of the model. Another limitation was the restricted release area of the five tagged dolphins off the coast of Galicia. It would be useful to increase the number of tags, but also to extend the tagging experiment area in Portuguese waters. The compromise between large-scale deployment and the specificity of local conditions, such as the currentology of the Galician rias, can be difficult to find [40–42]. As with the trajectory of carcass IV, the model can sometimes fail to predict the real stranding location. This may be due to a failure to account for local currents, gyres, coastal currents or frontal zone. Dolphins may also strand but remain undetected, be picked up by the tide and then strand in another location, a process that the model cannot predict. More generally, given the large number of drifts used, we consider that the impact of these drifts may be insignificant in the final estimates. However, further studies are needed to better understand why the model fails to predict the real stranding location with other tagged carcasses in the same area and to adjust the model if the problem is an environmental parameter. In addition, a consistent pattern emerges about the computational time: the trajectory calculation time increases with greater bathymetry accuracy. Moreover, increasing the resolution does not lead to a improvement in the accuracy of the predicted versus actual stranding locations for all individuals. Furthermore, a high level of precision does not contribute to improved accuracy in the distance between the predicted and actual stranding for all individuals (Table 1). This can be explained by the fact that the continental shelf to the west of the Iberian Peninsula is very narrow, between 30 km and 50 km wide, with a shelf break depth of about 160 m [37, 43]. When bathymetry is less than 100 m, its influence on drift currents becomes more significant. As a result, the use of bathymetry with a resolution of 4 arc-minutes would be sufficient to model the drift of small cetaceans.

The movement of an object drifting on the sea surface is mainly influenced by several forces acting on its surface: wind, currents and waves. In our simulations, wind and surface currents are systematically considered. The surface currents generated by waves is known as the Stokes drift [44]. Stokes drift can be caused by wind seas (waves generated by the local wind) and swell (waves that are no longer under the direct influence of the wind that generated them). Only waves generated by the local wind are taken into account in MOTHY. Simulations of the effect of swell on oil spill drift have given mixed results: for example, they were less accurate for the Erika drift but showed an improvement for the Prestige accident [26, 45]. In addition, Dagestad and Röhrs [46] have shown that incorporating Stokes drift into a drift model improves predictions for fully submerged objects, but has a limited impact for objects exposed to the surface wind. Thus, Stokes drift is therefore partially integrated into MOTHY with the sea wind, while swell is neglected as it is considered to have less impact on dolphin carcasses that are not fully submerged.

A number of methodologies have been devised to ascertain the provenance of megafauna found stranded, with a view to elucidating the observed trends [47]. The initial approaches were developed in the context of oil spills pollution, which primarily impacted seabirds. The aim of these studies was to release drifters or tagged animals at sea in order to estimate the proportion of oiled seabirds that died at sea and were subsequently found stranded [48, 49]. In more recent times, these approaches have also been employed to assess the significance of megafauna mortality, such as turtles [50–52], seabirds [53–55], otters [56, 57] and cetaceans [24, 31, 58]. Hydrodynamic models, such as MOTHY, which is based on the drift of water masses, are commonly used for simulating the movement of carcass at sea. Haelters et al.(2006) used a hydrodynamic drift model to determine the origin of porpoises stranded along the Belgian coast [59]. For sea turtle strandings, several approaches have been investigated. In 2006, Hart et al. used drift and currents models for their studies [60]. Later, in 2013, Nero et al. used an AMSEAS (American SEAS) hydrodynamic model based on the Navy Coastal Ocean Model [61]. Santos et al.(2018) used the Ichthyop software, originally designed for ichthyoplankton drift, to model sea turtle drift [51]. Finally, in 2019, Liu et al. applied an advanced community ocean model for the same purpose [62].

However, many of these models primarily take into account the movement of particles in the water mass, often neglecting or insufficiently considering the influence of wind on the emerging part of drifting objects. This factor can significantly impact drift trajectories, especially depending on the species under study. Regarding the drift of small cetacean carcasses, immersion rate is one of the most sensitive parameters affecting carcass drift simulations [24]. The utilisation of an object drift model, such as MOTHY, allows for the dimensions of the object in question to be taken into account, thereby improving drift predictions in terms of the wind and surface currents generated. in addition, the model can be employed to apply buoyancy parameters, which exert a considerable influence on drift speed [24].

Whatever model type is used, they all rely on the same basic parameters, namely wind and surface currents. Other parameters may be added depending on the area, such as tidal fluctuations [51, 63], wave strength or water temperature [62]. Tidal fluctuations were not accounted for in this study, as the tidal model for the area has not yet been developed by Météo France and integrated into MOTHY. However, it would be valuable to test the influence of this parameter once the model becomes available, to assess whether tides significantly impact drift trajectories in this area.

The decision to use the deterministic method rather than the probabilistic one is an advantageous in terms of population management. The management unit area for the North-East Atlantic population of common dolphins comprises OSPAR Regions II (Greater North Sea), III (Celtic Sea) and IV (Bay of Biscay and Iberian coast) [64]. In the Bay of Biscay, the drift model utilizes a deterministic calculation method to assess bycatch mortality. Adopting a similar approach enables the incorporation of mortality estimates for the Iberian coast within the OSPAR Region IV management unit. This allows for more accurate estimates of bycatch mortality at the population level based on strandings.

The modelling of all tagged dolphins showed that the majority of those found stranded were predicted to be stranded. Of the five animals found stranded, only one was not predicted to be stranded. In addition to these four animals, a further six carcasses were predicted to be stranded. However, the stranding network did not detect all tagged animals as stranded. This could be explained by the likely high proportion of dolphin that float. The most recent estimate of the proportion of floating carcasses is 24% CI[17;32] in the Bay of Biscay [65]. The remainder sank and were consequently lost for the stranding process. It is possible that these six carcasses had sunk and therefore could not get stranded. A large number of tagged carcasses would be needed to determine a rate specific to Iberian waters. Another explanation could be the advanced state of decomposition of the carcasses. In a highly decomposed state, the tag may likely have separated from the animal’s body preventing its proper identification. Alternatively, as mentioned above, dolphins carcasses may also strand but remain undetected, be picked up by the tide.

Reverse drift models can be used to retrace the trajectory of a carcass from the stranding to the site of death. Identifying areas of mortality is essential for the conservation of vulnerable species, as it allows the identification of areas and potential conditions that may be detrimental to specific populations. This information is of the utmost importance if management measures are to be implemented. For example, in Cape Cod Bay, Massachusetts, the study of Kemp’s ridley turtle (*Lepidochelys kempii*) strandings revealed a possible link with cold temperatures that the individuals could not tolerate [62]. Similarly, a contaminated area with botulinum toxin was identified in northern Lake Michigan based on common loon (*Gavia immer*) strandings [54]. In addition, estimating areas of higher mortality can highlight the overlap between megafauna occurence areas and fishing activities, as has been observed for sea turtles in Virginia’s Chesapeake Bay [51], as well as for small cetaceans and fishing efforts in the Bay of Biscay [63].

This study identifies key parameters for adapting a small cetacean drift model to new regions. Initial work can begin with a limited number of tags and later on, it can be refined by continuing the tagging of bycaught dolphins. The model is used to estimate mortality from strandings using reverse drift calculations. When applied to a management unit like the North-East common dolphin, this approach improves population-level mortality estimates by using standardized indicators and methodologies. This work can be also applied for other species of cetacean in wider distribution areas, supporting global assessment strategies and management.

## References

[1] Hammond PS, Macleod K, Berggren P, Borchers DL, Burt L, Cañadas A, et al. Cetacean abundance and distribution in European Atlantic shelf waters to inform conservation and management. Biological Conservation. 2013;164:107–122. doi: 10.1016/j.biocon.2013.04.010

[2] Moore SE. Marine mammals as ecosystem sentinels. Journal of Mammalogy. 2008;89(3):534–540. doi: 10.1644/07-MAMM-S-312R1.1

[3] Lebreton JD, Burnham KP, Clobert J, Anderson DR. Modeling survival and testing biological hypotheses using marked animals: a unified approach with case studies. Ecological monographs. 1992;62(1):67–118. doi: 10.2307/2937171

[4] Dodge Y. The Oxford dictionary of statistical terms. OUP Oxford; 2006.

[5] Magera AM, Mills Flemming JE, Kaschner K, Christensen LB, Lotze HK. Recovery trends in marine mammal populations. PloS one. 2013;8(10):e77908. doi: 10.1371/journal.pone.0077908 24205025 PMC3813518

[6] Wang IM, Michalak NM, Ackerman JM. Life History Strategies. In: Shackelford TK, Weekes-Shackelford VA, editors. Encyclopedia of Evolutionary Psychological Science. Cham: Springer International Publishing; 2021. p. 4560–4569.

[7] Read AJ. The looming crisis: interactions between marine mammals and fisheries. Journal of Mammalogy. 2008;89(3):541–548. doi: 10.1644/07-MAMM-S-315R1.1

[8] Read AJ, Drinker P, Northridge S. Bycatch of Marine Mammals in U.S. and Global Fisheries: Bycatch of Marine Mammals. Conservation Biology. 2006;20(1):163–169. doi: 10.1111/j.1523-1739.2006.00338.x 16909669

[9] Reeves RR, Berggren P, Crespo EA, Gales N, Northridge SP, di Sciara GN, et al. Global priorities for reduction of cetacean bycatch. World Wildlife Fund. 2005;.

[10] Hammond P, Bearzi G, Bjørge A, Forney K, Karczmarski L, Kasuya T, et al. Phocoena phocoena. The IUCN Red List of Threatened Species 2008: e. T17027A6734992;

[11] Mangel JC, Alfaro-Shigueto J, Van Waerebeek K, Cáceres C, Bearhop S, Witt MJ, et al. Small cetacean captures in Peruvian artisanal fisheries: high despite protective legislation. Biological Conservation. 2010;143(1):136–143. doi: 10.1016/j.biocon.2009.09.017

[12] Rojas-Bracho L, Gulland F, Smith CR, Taylor B, Wells R, Thomas P, et al. A field effort to capture critically endangered vaquitas Phocoena sinus for protection from entanglement in illegal gillnets. Endangered Species Research. 2019;38:11–27. doi: 10.3354/esr00931

[13] Perrin WF. Common Dolphin: *Delphinus delphis*. In: Encyclopedia of marine mammals. Elsevier; 2018. p. 205–209.

[14] Evans PG, Teilmann J. ASCOBANS/HELCOM small cetacean population structure workshop. Bonn, Germany: ASCOBANS. 2009;.

[15] Murphy S, Evans PGH, Pinn E, Pierce GJ. Conservation management of common dolphins: Lessons learned from the North‐East Atlantic. Aquatic Conservation: Marine and Freshwater Ecosystems. 2021;31(S1):137–166. doi: 10.1002/aqc.3212

[16] Caurant F, Chouvelon T, Lahaye V, Mendez-Fernandez P, Rogan E, Spitz J, et al. The use of ecological tracers for discriminating dolphin population structure: the case of the short-beaked common dolphin *Delphinus delphis* in European Atlantic waters. International Whaling Commission: Madeira. 2009;.

[17] ICES. 2024. Working Group on Bycatch of Protected Species (WGBYC). ICES Scientific Reports. 5:111. 334 pp.

[18] Taylor N, Authier M, Banga R, Genu M, Gilles A. Marine Mammal By-catch;2022 In: OSPAR, 2023: The 2023 Quality Status Report for the Northeast Atlantic. OSPAR Commission, London. Available at: https://oap.ospar.org/en/ospar-assessments/quality-status-reports/qsr2023/indicator-assessments/marine-mammal-bycatch.

[19] Hammond P, Lacey C, Gilles A, Viquerat S, Börjesson P, Herr H, et al. Estimates of cetacean abundance in European Atlantic waters in summer 2016 from the SCANS-III aerial and shipboard surveys. Wageningen Marine Research; 2017.

[20] Gilles A, Authier M, Ramirez-Martinez N, Araujo H, Blanchard A, Carlstrom J, et al. Estimates of cetacean abundance in European Atlantic waters in summer 2022 from the SCANS-IV aerial and shipboard surveys. University of Veterinary Medicine Hannover; 2023.

[21] Mangi SC, Dolder PJ, Catchpole TL, Rodmell D, de Rozarieux N. Approaches to fully documented fisheries: practical issues and stakeholder perceptions. Fish and Fisheries. 2015;16(3):426–452. doi: 10.1111/faf.12065

[22] Moore JE, Heinemann D, Francis TB, Hammond PS, Long KJ, Punt AE, et al. Estimating Bycatch Mortality for Marine Mammals: Concepts and Best Practices. Frontiers in Marine Science. 2021;8. doi: 10.3389/fmars.2021.752356

[23] Michelin M, Elliott M, Bucher M, Zimring M, Sweeney M. Catalyzing the growth of electronic monitoring in fisheries. California Environmental Associates and the Nature Conservancy. 2018;.

[24] Peltier H, Dabin W, Daniel P, Van Canneyt O, Domérus G, Huon M, et al. The significance of stranding data as indicators of cetacean populations at sea: Modelling the drift of cetacean carcasses. Ecological Indicators. 2012;. doi: 10.1016/j.ecolind.2011.11.014

[25] Daniel P, Jan G, Cabioc’h F, Landau Y, Loiseau E. Drift Modeling of Cargo Containers. Spill Science & Technology Bulletin. 2002;7(5-6):279–288. doi: 10.1016/S1353-2561(02)00075-0

[26] Daniel P, Josse P, Dandin P, Lefevre JM, Lery G, Cabioch F, et al. Forecasting the Prestige oil spills. In: Proceedings of the Interspill 2004 conference, Trondheim, Norway; 2004.

[27] Peltier H, Baagøe HJ, Camphuysen KC, Czeck R, Dabin W, Daniel P, et al. The stranding anomaly as population indicator: the case of harbour porpoise Phocoena phocoena in North-Western Europe. PLoS One. 2013;8(4):e62180. doi: 10.1371/journal.pone.0062180 23614031 PMC3632559

[28] ICES. 2019. Working group on marine mammal ecology (WGMME). 2019; ICES Scientific Reports. 1:22. 131 pp.

[29] ICES. 2020. Workshop on fisheries Emergency Measures to minimize BYCatch of short-beaked common dolphins in the Bay of Biscay and harbour porpoise in the Baltic Sea (WKEMBYC). ICES Scientific Reports. 2:43. 295 pp.

[30] ICES. 2023. Workshop on mitigation measures to reduce bycatch of short-beaked common dolphins in the Bay of Biscay (WKEMBYC2). 2023; ICES Scientific Reports. 5:3. 96 pp.

[31] Peltier H, Authier M, Deaville R, Dabin W, Jepson PD, van Canneyt O, et al. Small cetacean bycatch as estimated from stranding schemes: The common dolphin case in the northeast Atlantic. Environmental Science & Policy. 2016;63:7–18. doi: 10.1016/j.envsci.2016.05.004

[32] Goetz S, Read FL, Santos MB, Pita C, Pierce GJ. Cetacean–fishery interactions in Galicia (NW Spain): results and management implications of a face-to-face interview survey of local fishers. ICES Journal of Marine Science. 2014;71(3):604–617. doi: 10.1093/icesjms/fst149

[33] Goetz S, Read FL, Ferreira M, Portela JM, Santos MB, Vingada J, et al. Cetacean occurrence, habitat preferences and potential for cetacean–fishery interactions in Iberian Atlantic waters: results from cooperative research involving local stakeholders. Aquatic Conservation: Marine and Freshwater Ecosystems. 2015;25(1):138–154. doi: 10.1002/aqc.2481

[34] López A, Santos MB, Pierce GJ, González AF, Valeiras X, Guerra A. Trends in strandings and by-catch of marine mammals in north-west Spain during the 1990s. Journal of the Marine Biological Association of the United Kingdom. 2002;82(3):513–521. doi: 10.1017/S0025315402005805

[35] López A, Pierce GJ, Santos MB, Gracia J, Guerra A. Fishery by-catches of marine mammals in Galician waters: results from on-board observations and an interview survey of fishermen. Biological Conservation. 2003;111(1):25–40. doi: 10.1016/S0006-3207(02)00244-6

[36] Fernández-Contreras MM, Cardona L, Lockyer CH, Aguilar A. Incidental bycatch of short-beaked common dolphins (Delphinus delphis) by pairtrawlers off northwestern Spain. ICES Journal of Marine Science. 2010;67(8):1732–1738. doi: 10.1093/icesjms/fsq077

[37] Montadert L, Winnock E, Deltiel J, Grau G. Continental margins of Galicia-Portugal and Bay of Biscay. In: The geology of continental margins. Springer; 1974. p. 323–342.

[38] Pérez-Alberti A, de Compostela S, Pires A, Freitas L, Rodrigues C, Chaminé HI. GIS Mapping and Shoreline Change Analysis along the Rocky Coast of Galicia (NW Spain): Preliminary Approach. Proceeding of the International Coastal Engineering Conference (ICE). 2011;.

[39] Daniel P, Josse P, Dandin P. Further improvement of drift forecast at sea based on operational oceanography systems. WIT Transactions on The Built Environment. 2005;78.

[40] Alvarez-Salgado XA, Rosón G, Pérez FF, Pazos Y. Hydrographic variability off the Rías Baixas (NW Spain) during the upwelling season. Journal of Geophysical Research. 1993;98(C8):14447. doi: 10.1029/93JC00458

[41] Alvarez I, deCastro M, Gomez-Gesteira M, Prego R. Inter- and intra-annual analysis of the salinity and temperature evolution in the Galician Rías Baixas–ocean boundary (northwest Spain). Journal of Geophysical Research: Oceans. 2005;110(C4). doi: 10.1029/2004JC002504

[42] Fiúza AFG. Upwelling Patterns off Portugal. In: Suess E, Thiede J, editors. Coastal Upwelling Its Sediment Record: Part A: Responses of the Sedimentary Regime to Present Coastal Upwelling. NATO Conference Series. Boston, MA: Springer US; 1983. p. 85–98.

[43] Ravara A, Moreira MH. Polychaeta (Annelida) from the continental shelf off Aveiro (NW Portugal): Species composition and community structure. Check List. 2013. doi: 10.15560/9.3.533

[44] Stokes GG. On the theory of oscillatory waves. Trans Cam Philos Soc. 1847;8:441–455.

[45] Daniel P, Marty F, Josse P, Skandrani C, Benshila R. Improvement of Drift Calculation in Mothy Operational Oil Spill Prediction System. International Oil Spill Conference Proceedings. 2003;2003(1):1067–1072. doi: 10.7901/2169-3358-2003-1-1067

[46] Dagestad KF, Röhrs J. Prediction of ocean surface trajectories using satellite derived vs. modeled ocean currents. Remote sensing of environment. 2019;223:130–142. doi: 10.1016/j.rse.2019.01.001

[47] da Cunha Ramos HG, Colosio AC, Marcondes MCC, Schmidt AJ, Ghisolfi RD, Michalski BE, et al. An Overview of Forensic Ecology applied for marine megafauna conservation. Forensic Science International: Animals and Environments. 2024; p. 100085.

[48] Jones PH, Monnat JY, Cadbury C, Stowe T. Birds oiled during the Amoco Cadiz incident—an interim report. Marine pollution bulletin. 1978;9(11):307–310. doi: 10.1016/0025-326X(78)90256-4

[49] Hlady DA, Burger AE. Drift-block experiments to analyse the mortality of oiled seabirds off Vancouver Island, British Columbia. Marine Pollution Bulletin. 1993;26(9):495–501. doi: 10.1016/0025-326X(93)90466-W

[50] Koch V, Peckham H, Mancini A, Eguchi T. Estimating at-sea mortality of marine turtles from stranding frequencies and drifter experiments. PloS one. 2013;8(2):e56776. doi: 10.1371/journal.pone.0056776 23483880 PMC3577704

[51] Santos BS, Friedrichs MA, Rose SA, Barco SG, Kaplan DM. Likely locations of sea turtle stranding mortality using experimentally-calibrated, time and space-specific drift models. Biological Conservation. 2018;226:127–143. doi: 10.1016/j.biocon.2018.06.029

[52] Cook M, Reneker JL, Nero RW, Stacy BA, Hanisko DS, Wang Z. Use of drift studies to understand seasonal variability in sea turtle stranding patterns in Mississippi. Frontiers in Marine Science. 2021;8:659536. doi: 10.3389/fmars.2021.659536

[53] Wiese FK. Sinking rates of dead birds: improving estimates of seabird mortality due to oiling. Marine Ornithology. 2003;31(1):65–70. Available at: https://digitalcommons.usf.edu/marine_ornithology/vol31/iss1/9

[54] Kenow KP, Ge Z, Fara LJ, Houdek SC, Lubinski BR. Identifying the origin of waterbird carcasses in Lake Michigan using a neural network source tracking model. Journal of Great Lakes Research. 2016;42(3):637–648. doi: 10.1016/j.jglr.2016.02.014

[55] Martin N, Varela VW, Dwyer FJ, Tuttle P, Ford RG, Casey J. Evaluation of the fate of carcasses and dummies deployed in the nearshore and offshore waters of the northern Gulf of Mexico. Environmental monitoring and assessment. 2019;191:1–13. doi: 10.1007/s10661-019-7923-0 PMC7078152 32185518

[56] Degange AR, Doroff AM, Monson DH. Experimental recovery of sea otter carcasses at Kodiak Island, Alaska, following the Exxon Valdez oil spill. Marine mammal science. 1994;10(4):492–496. doi: 10.1111/j.1748-7692.1994.tb00509.x

[57] Young C, Eguchi T, Ames JA, Staedler M, Hatfield BB, Harris M, et al. Drift and beaching patterns of sea otter carcasses and car tire dummies. Marine Mammal Science. 2019;35(4):1512–1526. doi: 10.1111/mms.12609

[58] da Cunha Ramos HG, Colosio AC, Marcondes MCC, Fontes FC, Dapper CG, de Oliveira Campos R, et al. Carcass non-recovery rate of franciscana dolphin (Pontoporia blainvillei), calibrated with a drift mark-recapture study at FMA Ia, Brazil. Latin American Journal of Aquatic Mammals. 2022;17(2):93–104. doi: 10.5597/lajam00288

[59] Haelters J, Jauniaux T, Kerckhof F, Ozer J, Scory S. Using models to investigate a harbour porpoise bycatch problem in the southern North Sea-eastern Channel in spring 2005. Figshare. 2006;.

[60] Hart KM, Mooreside P, Crowder LB. Interpreting the spatio-temporal patterns of sea turtle strandings: going with the flow. Biological Conservation. 2006;129(2):283–290. doi: 10.1016/j.biocon.2005.10.047

[61] Nero RW, Cook M, Coleman AT, Solangi M, Hardy R. Using an ocean model to predict likely drift tracks of sea turtle carcasses in the north central Gulf of Mexico. Endangered Species Research. 2013;21(3):191–203. doi: 10.3354/esr00516

[62] Liu X, Manning J, Prescott R, Page F, Zou H, Faherty M. On simulating cold-stunned sea turtle strandings on Cape Cod, Massachusetts. PloS one. 2019;14(12):e0204717. doi: 10.1371/journal.pone.0204717 31800593 PMC6892539

[63] Peltier H, Authier M, Dabin W, Dars C, Demaret F, Doremus G, et al. Can modelling the drift of bycaught dolphin stranded carcasses help identify involved fisheries? An exploratory study. Global Ecology and Conservation. 2020;21:e00843. doi: 10.1016/j.gecco.2019.e00843

[64] ICES. 2014. Report of the Working Group on Marine Mammal Ecology (WGMME). 10–13 March 2014, Woods Hole, Massachusetts, USA. ICES CM 2014/ACOM:27. 232 pp.

[65] Dars C., Méheust E., Genu M., Méndez-Fernandez P., Peltier H., Wund S., et al. 2023. Les échouages de mammifères marins sur le littoral français en 2022.; Rapport scientifique de l’Observatoire Pelagis, La Rochelle Université et CNRS. 49 pages.

